# A cost-outcome assessment of dog deworming for echinococcosis control in the Xizang Autonomous Region, China, 2016–2022

**DOI:** 10.1371/journal.pntd.0014580

**Published:** 2026-08-07

**Authors:** Mingzhe Jiang, Quzhen Gongsang, Zhiyi Wang, Jiaxi Lei, Liying Wang

**Affiliations:** 1 National Institute of Parasitic Diseases, Chinese Center for Disease Control and Prevention; Chinese Center for Tropical Diseases Research; National Key Laboratory of Intelligent Tracking and Forecasting for Infectious Diseases; NHC Key Laboratory of Parasite and Vector Biology; WHO Collaborating Centre for Tropical Diseases; National Center for International Research on Tropical Diseases, Ministry of Science and Technology, Shanghai, China; 2 Tibet Center for Disease Control and Prevention; NHC Key Laboratory of Echinococcosis Prevention and Control, Lhasa, China; Cyprus International University: Uluslararasi Kibris Universitesi, CYPRUS

## Abstract

**Background:**

Xizang is the most endemic region of echinococcosis in China. Dog deworming is the most important measure in Central Government’s Transfer Payment Project for Echinococcosis Control. Yet the economic performance of dog deworming across diverse settings in Xizang has not been systematically assessed. To address this gap, we examined the cost of dog deworming and assessed its outcome at county-level in the Xizang Autonomous Region (Xizang).

**Methods:**

We collected data on dog management and deworming from 74 counties in Xizang from 2016 to 2022. Canine infection rate was used as the key indicator to assess the outcome of dog deworming. Statistical analyses were conducted to examine temporal changes in canine infection rate and related indicators, while spatial analyses were performed to compare county-level differences in cost, outcome, and cost-outcome. County-level cost-outcome analysis was further conducted to evaluate the cost required for every percentage-point reduction in the canine infection rate.

**Results:**

The canine infection rate showed a 91.1% reduction, from 5.63% in 2016 to 0.50% in 2022. The outcome of dog deworming displayed significant spatial variation. Canine infection rate decreased in 67 of 74 counties, and the reduction magnitude exceeded 80% in 46 counties. However, 7 counties showed a slight increase. Considerable regional heterogeneity in cost-outcome was observed: 44 of 74 counties required less than 50,000 CNY for each percentage point reduction in canine infection rate, including 19 counties below 10,000 CNY, whereas 6 counties exceeded 200,000 CNY per percentage point reduction.

**Conclusion:**

Dog deworming, as part of a sustained integrated control program, was associated with substantial reductions in canine Echinococcus infection in Xizang and showed favorable programmatic cost-outcome performance. As a major and key control measure, dog deworming should be sustained and consistently implemented. Particular attention should be paid to counties where the effect was not obvious or where infection rate increased, and the underlying reasons should be investigated to ensure that dog deworming is fully implemented. In addition, dog registration and tethering, stray dog sheltering, and proper disposal of canine feces should be further strengthened to improve the efficiency of source-control measures.

## Author summary

Echinococcosis, also known as hydatidosis, is a serious parasitic infection that affects both animals and humans. In China, Xizang has the highest burden of the disease. Dogs play a key role in spreading echinococcosis, and regular dog deworming is a critical measure to break the transmission chain. In this study, we analyzed data from 74 counties in Xizang from 2016 to 2022 to understand how well dog deworming programs work and to evaluate their cost-outcome performance. We found that canine infection rate has fallen sharply over the years, showing clear progress in echinococcosis control. However, cost, outcome, and cost-outcome varied across counties. While many counties achieved favorable results, some areas had high cost but relatively limited improvement. These findings show that dog deworming, as part of an integrated control program, was associated with reductions in canine Echinococcus infection in Xizang. This study provides practical evidence for improving the implementation efficiency of dog deworming in Xizang and may also offer useful reference for other endemic areas.

## 1. Introduction

Echinococcoses are serious zoonotic parasitic disease caused by the larval stages of *Echinococcus spp*. [1]*.* The primary causative agents of echinococcoses in China are *Echinococcus granulosus sensu lato* (causing cystic echinococcosis, CE) and *Echinococcus multilocularis* (causing alveolar echinococcosis, AE) [2,3]. Echinococcosis presents a severe public health challenge in China, which reports the world's highest prevalence. With over 25 thousand patients and a prevalence of 0.05%, the disease's impact is profound on a global scale: China contributes to 40% of global CE cases and an overwhelming 91% of AE cases. Consequently, the associated disease burden in China is estimated at 322,400 disability adjusted life years (DALYs), representing 40% and 95% of the global DALYs for CE and AE, respectively [4–7]. It poses considerable threats against human health as well as socioeconomic development [8]. In China, echinococcosis is endemic over 370 counties from 9 provinces or autonomous regions [9]. Xizang is the most severely affected. Xizang comprises seven prefecture-level cities and 74 counties/districts, all of which are endemic counties for echinococcosis. Xizang exhibits the highest human prevalence of echinococcosis in the country, at 1.66% [10]. Furthermore, it bears a disproportionately high disease burden, accounting for an estimated 99,700 DALYs [4].

*Echinococcus granulosus sensu lato* is primarily transmitted through the domestic animal cycle (livestocks and dogs). *Echinococcus multilocularis* is mainly maintained in a wildlife cycle involving rodents, lagomorphs, and canids. However, in China, dogs, especially stray dogs, play a primary role in the transmission of AE. In China, dogs are the definitive hosts for both species of *Echinococcus* and thus the main reservoir for their transmission [11–13]. Xizang has a large dog population and a high risk of echinococcosis transmission. Most herdsmen families in Xizang keep at least one dog, and Buddhist monks often care for large numbers of ownerless stray dogs [14]. Under these conditions, dogs constitute an important source of infection [15]. Therefore, within the Central Government’s Transfer Payment Project for Echinococcosis Control, dog deworming has been adopted as the most important source-control measure. At the same time, other complementary interventions, such as dog population reduction, stray dog sheltering, and related measures, have also been implemented. With the implementation of the project, the local government advocated strict restrictions on the number of dogs, and the number of dogs continued to decrease. The canine infection rate in Xizang decreased significantly from 7.3% in 2016 to 1.7% in 2019 [16]. Therefore, regular dog deworming remains a core source-control measure for echinococcosis prevention and control [17–19].

Therefore, we systematically collected data from 2016 to 2022 to describe the temporal trends and spatial distribution of canine infection rate, and to characterize the cost and outcome of dog deworming. The findings are intended to provide evidence and practical guidance for improving the implementation of dog deworming in Xizang.

## 2. Materials and methods

### 2.1. Ethics approval and consent to participate

Not applicable.

### 2.2. Consent for publication

Not applicable.

### 2.3. Data sources

Data regarding dog management in Xizang's 74 counties from 2016 to 2022 were obtained and aggregated from the annual reporting system of the Xizang Autonomous Region Center for Disease Control and Prevention (CDC). The data included: registered dog numbers, stray dog numbers, frequency of dog deworming, frequency of wild canids deworming, and the examination results of dog fecal samples, annual dog deworming expenditure data from 74 counties in Xizang.

Registered dog numbers were obtained through routine household-level registration, whereby grassroots staff registered each owned dog according to program requirements. Stray dog numbers were counted and recorded at the village level. Both indicators were then aggregated from the village level to the township level, then to the county level, and finally compiled at the Xizang Autonomous Region CDC. Canine infection status was assessed using copro-ELISA as a standardized diagnostic method applied consistently across all counties during the study period. Considering the practical conditions of field surveillance, as well as the advantages of this method in terms of feasibility, convenience, and cost, copro-ELISA has been adopted as the routine surveillance method in the National Echinococcosis Surveillance Scheme (2020 Edition). Before being approved for routine surveillance use, the copro-ELISA assay was evaluated by the National Institute of Parasitic Diseases. The Echinococcus coproantigen detection kit used in this study had an evaluated sensitivity of 100% (95% CI: 92.86%–100%) and specificity of 100% (95% CI: 91.24%–100%). In addition, canine fecal samples in all counties were tested under unified technical requirements and standardized procedures, and relevant testing personnel received specialized technical training. In each county, 20 positive canine fecal samples and 20 negative canine fecal samples were retained for review, with all samples retained if fewer than 20 were available. These retained samples were required to be submitted to the Xizang Autonomous Region CDC within 3 months after completion of testing for confirmatory review, in order to improve the accuracy and reliability of the results.

### 2.4. Research methods

This study was an ecological observational analysis based on aggregated county-level surveillance and program cost data.

#### 2.4.1 Calculation of dog deworming cost.

Dog deworming cost was estimated based on the standard dosage of praziquantel for dog deworming as specified in the Technical Scheme for Echinococcosis Control (2019 Edition). The cost data used in this study were derived from the earmarked dog deworming module within the Central Government’s Transfer Payment Project for Echinococcosis Control. The unit price of praziquantel was obtained through the unified governmental procurement system of Xizang. Based on these standardized dosage requirements and administratively determined procurement prices, Xizang conducts annual accounting of dog deworming program expenditures for each county. The resulting cost estimates represent total program-level dog deworming cost and include expenditures related to antiparasitic drugs, personnel, and logistics, as reported through standardized administrative accounting procedures.

The dog deworming cost data integrity was checked, whereby missing or anomalous values were addressed. The data were then standardized and all costs were converted to a common unit (1 Yuan, CNY).

Analysis of the county-level data regarding dog deworming from 2016 to 2022 within the Xizang Autonomous Region was carried out using the variables: dog population, the number of registered dogs and the frequency of dog deworming from the data collected. *Dog management rate* was determined by dividing the number of registered dogs by the total dog population. *The average annual deworming frequency per dog* and *the deworming compliance rate* were calculated based on the frequency of deworming and the guidelines outlined in the “Technical Scheme for Echinococcosis Control (2019 Edition)” [20]. The deworming compliance rate = Number of Deworming Treatments / Number of Registered Dogs, and the deworming compliance rate was determined using 12 times per year as the standard.

The characteristics of dog deworming cost over the years and across regions were then analyzed. According to the changes in the total cost of dog deworming and canine infection rate in each county from 2016 to 2022, the cost and outcome of dog deworming in 74 counties in Xizang from 2016 to 2022 was systematically assessed. The one-sample Kolmogorov-Smirnov (K-S) test was used to determine if the infection rate had a normal distribution. Kruskal-Wallis (K-W) rank sum test was used to analyse differences in infection rate among the years. In addition, the Jonckheere-Terpstra (J-T) test was used to analyse the infection rate from 2016 to 2022. Since there were dynamics in the baseline infection rate, implying varying levels of difficulty in controlling the infections, a stratified analysis was carried out according to the 2016 infection rate.

ArcGIS 10.8 (Esri Inc., Redlands, CA, USA) was used to create the distribution map showing the dog deworming cost across counties. Spatial autocorrelation analysis was used to assess spatial dependence and reveal distribution patterns, with Z-scores indicating clustering (Z > 0: positive autocorrelation—similar values cluster; Z < 0: negative autocorrelation—dissimilar values disperse). Hotspot analysis was then used to identify statistically significant clusters of high- and low-cost values.

#### 2.4.2 Assessment of Dog Deworming Outcome.

Based on canine fecal examination data from 2016 to 2022, the annual and regional coprological positivity rates were calculated to determine the temporal trend of the overall positivity rate in Xizang. Spatial distribution analysis of the *positivity rate*, including spatial autocorrelation analysis, hot spot analysis, spatial cluster and outlier analysis, was carried out using ArcGIS 10.8. The statistical significance level was set at α = 0.05. The *outcome of dog deworming* was quantified based on change in the infection rate, defined as follows: Canine Infection Rate = Positive Number of Canine Fecal Samples / Number of canine fecal samples examined, Deworming Outcome (R) = Canine Infection Rate (2016) - Canine Infection Rate (2022). An R value > 0 indicates a reduction in canine infection rate, R = 0 indicates no net change, and R < 0 indicates an increase over the study period. Sample sizes varied across counties and years due to operational constraints. In this study, the canine infection rate was defined as the copro-ELISA positivity rate for canine *Echinococcus* infection, which is used as the routine surveillance indicator in the Central Government’s Transfer Payment Project for Echinococcosis Control. Following the diagnostic-performance adjustment framework described by Lewis and Torgerson, point-estimate correction was considered; because the evaluated sensitivity and specificity of the assay were both 100%, the adjusted infection rate was identical to the observed copro-ELISA positivity rate [21]. Although the evaluated point estimates of sensitivity and specificity were both 100%, the confidence intervals indicate residual diagnostic uncertainty. In addition, sample sizes varied across counties and years, and therefore sampling variability may have influenced county-level estimates, particularly in counties with low copro-ELISA positivity rates.

#### 2.4.3 Cost-outcome analysis of dog deworming.

In this study, a cost-outcome analysis was conducted from a program implementation perspective to compare the efficiency of dog deworming across counties. Program efficiency was summarized as the cost required to achieve a one percentage-point absolute reduction in canine infection rate. Total cost-outcome of dog deworming in Xizang was determined by using the *annual deworming cost* and dynamics in *canine infection rate* per county. The analysis was performed by calculating the cost required to achieve a one percent absolute decrease in the canine infection rate. Regional disparities in cost-outcome were assessed to determine areas with better as well as poorer cost-outcome.

In counties where deworming was regarded as “effective,” the cost-outcome was determined as follows: Cost-outcome = Total Dog Deworming Cost (2016–2022) / R. The result was expressed in units of 10,000 CNY per percentage point. Considering the *annual deworming cost* and dynamics in *infection rate* in each county, the cost for each percentage point reduction in the canine infection rate was used to assess and compare the overall cost-outcome of dog deworming across different regions within Xizang.

## 3. Results

### 3.1. Dog deworming cost

**Fig 1:**
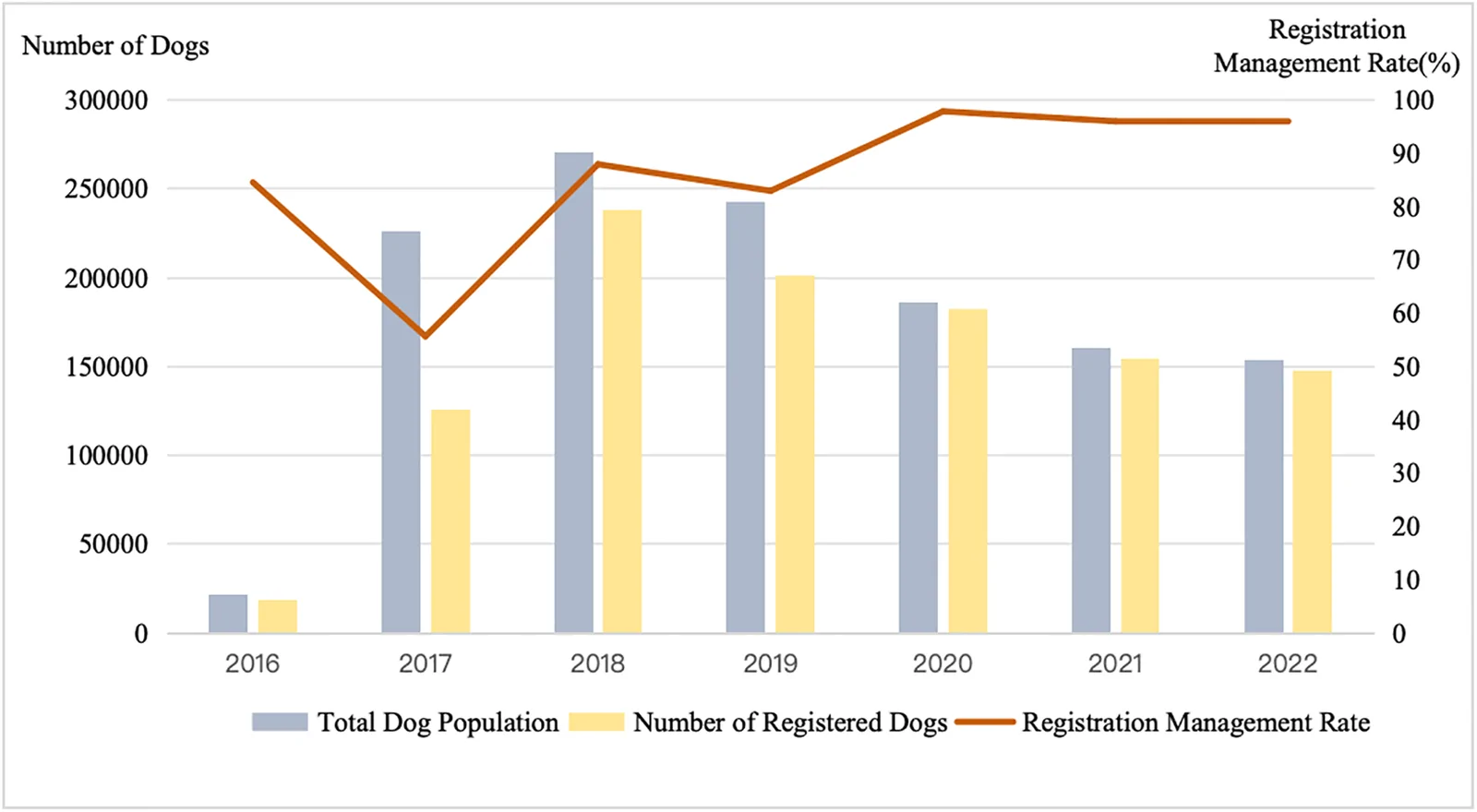
Dog Management Status in Xizang from 2016 to 2022.

Dog population and the number of registered dogs increased annually until 2018, peaking at 270,460 and 237,900, respectively. Thereafter, both decreased annually, reaching 154,000 and 148,000 by 2022. Meanwhile, the overall dog registration management rate increased steadily from 84.6% in 2016 to 96.2% in 2022. (Fig 1).

The total cost of dog deworming in the Xizang from 2016 to 2022 was 10.9546 million CNY. The cost displayed a trend of initial increase followed by a subsequent decrease. The lowest cost was recorded in 2016 (0.1509 million CNY), which increased sharply to 2.7591 million CNY in 2019—approximately a 17-fold increase compared to 2016—and then decreased to 1.3365 million CNY in 2022. The deworming compliance rate showed a trend of increasing first and then decreasing. The deworming compliance rate reached to 99.65% in 2020 but decreased to 77.20% in 2022 (Table 1).

**Table 1:** Dog Deworming Implementation in Xizang from 2016 to 2022.

Year	Total dog population (10,000)	Number of Registered Dogs (10,000)	Number of Deworming Treatments (10,000)	Average Deworming Frequency per Dog	Cost (10,000 CNY)	Deworming Compliance Rate (%)
2016	2.20	1.86	15.48	8.33	15.09	69.44
2017	22.64	12.61	37.55	2.94	36.61	24.49
2018	27.05	23.79	251.93	10.59	245.63	88.24
2019	24.27	20.14	282.98	10.31	275.91	85.90
2020	18.60	18.21	217.73	11.96	212.28	99.65
2021	16.08	15.44	181.31	11.74	176.78	97.87
2022	15.38	14.80	137.08	9.26	133.65	77.20
Total	126.21	107.79	1124.05	10.42	1095.95	86.80

**Fig 2:**
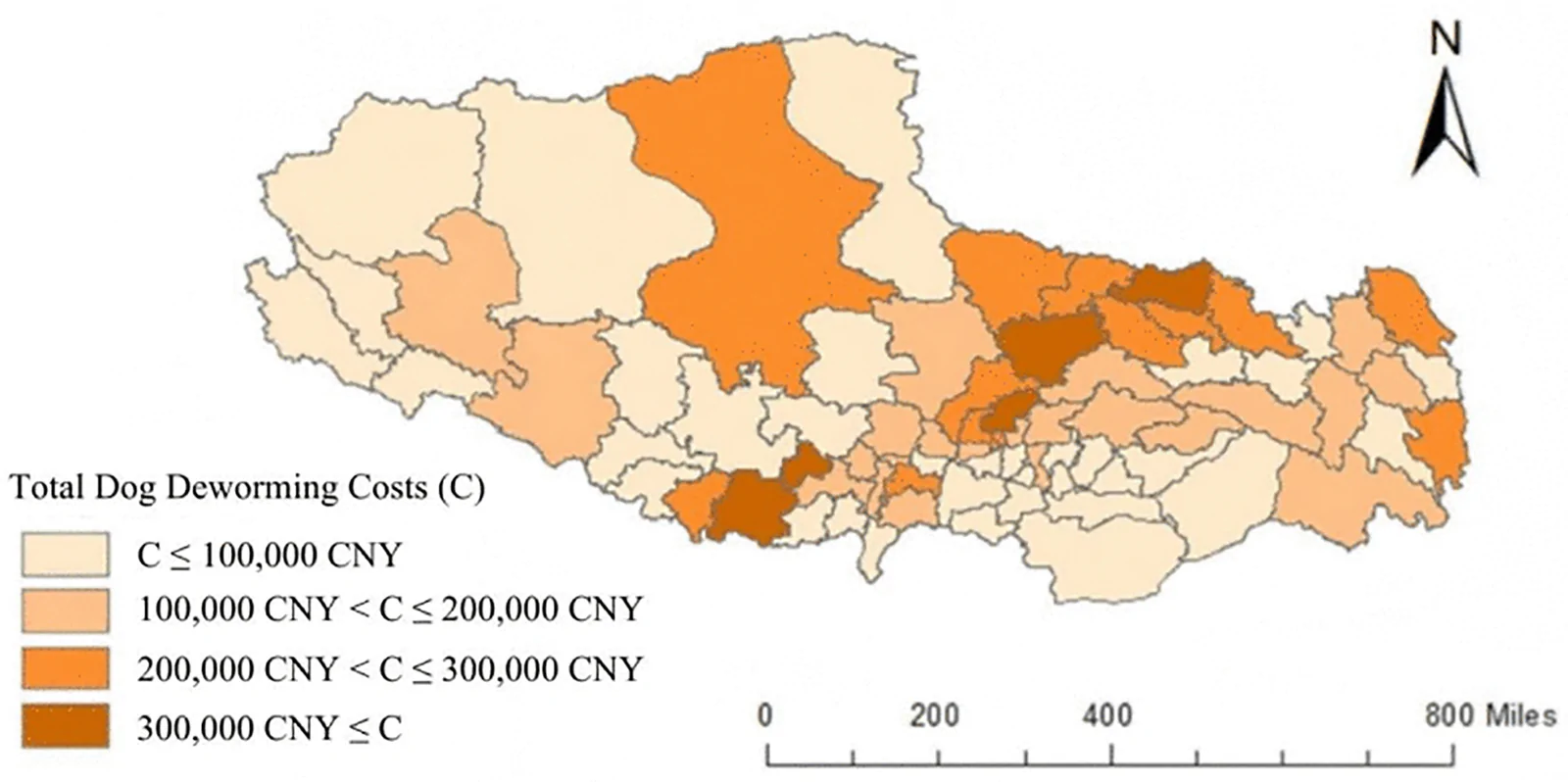
Total dog deworming cost across 74 counties in Xizang, 2016-2022. The base layer is from *https://www.resdc.cn/DOI/DOI.aspx?DOIID=120* with credit to Resource and Environment Science and Data Center.

The total cost of dog deworming in 74 counties was 10.95 million CNY, with an average investment of 0.148 million CNY per county. Substantial spatial disparities in deworming costs were observed across counties (Fig 2). Among the 74 counties, only 5 counties reported total cost exceeding 0.3 million CNY (6.76%), with the highest expenditures in Lhünzhub County of Lhasa City (0.99 million CNY) and Lhaze County of Xigazê City (0.96 million CNY), both significantly above the regional average. 13 counties spent between 0.2-0.3 million CNY (17.57%), 21 counties between 0.1-0.2 million CNY (28.38%), and 35 counties less than 0.1 million CNY (47.29%)—mainly located in Lhoka, Xigazê, and Ngari Prefecture. The lowest costs were observed in Cona County of Lhoka City (0.013 million CNY), Gar County of Ngari Prefecture (0.019 million CNY), and Gyirong County of Xigazê City (0.020 million CNY).

### 3.2. Deworming outcome

**Fig 3:**
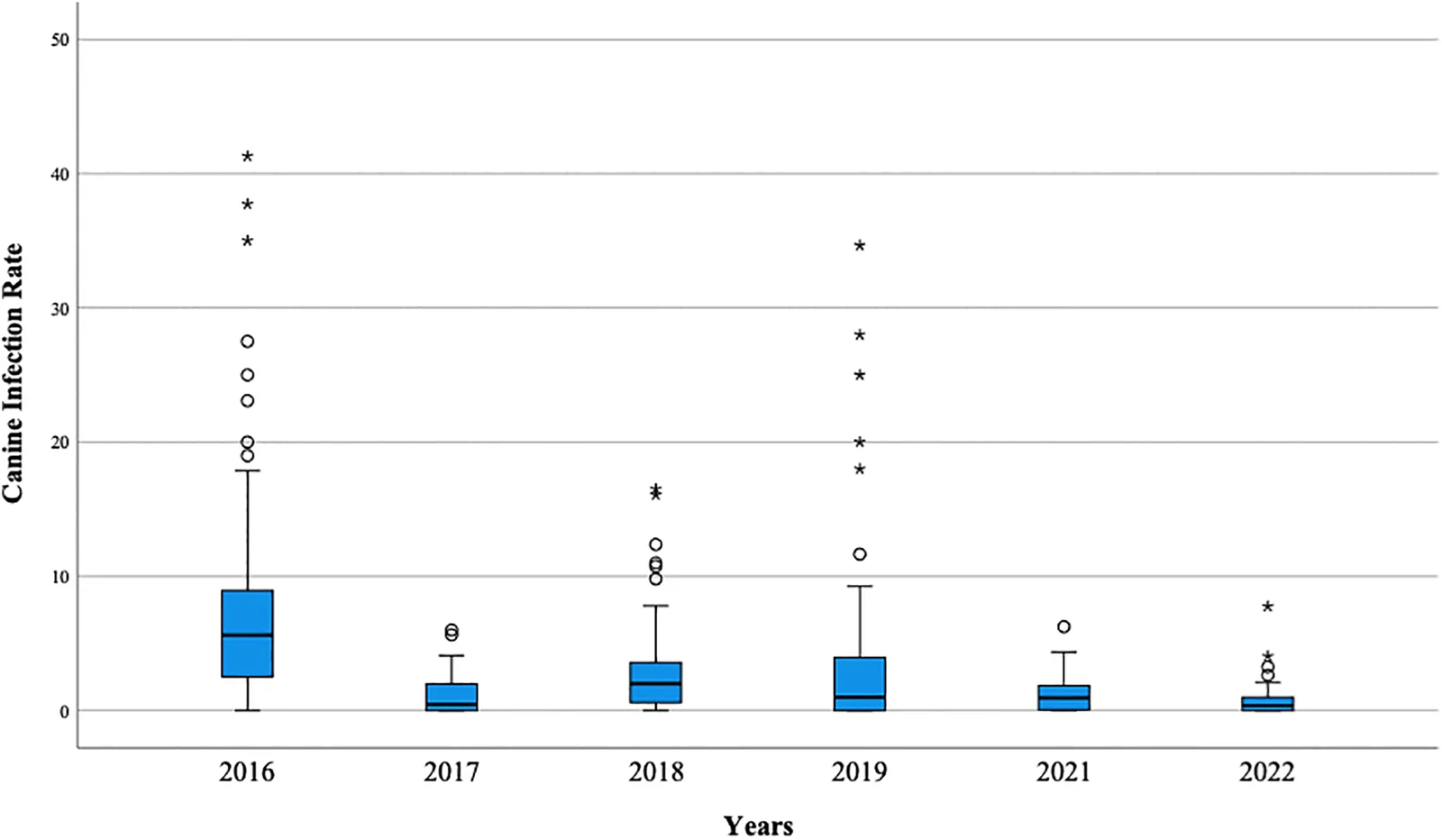
Canine infection rate in Xizang from 2016 to 2022.

Overall, a significant decrease in Echinococcus infection in dogs was observed from 2016 to 2022. Across 74 counties, infection rate ranged from 0.00% to 41.3%, with the highest infection rate of 41.3% in Bachen County of Nagqu City, in 2016. The K-W rank sum test shows significant inter-annual differences (H = 19.650, P < 0.01). A J-T trend test shows a significantly decreasing trend over these years (Z = -4.072, P < 0.01). The total canine infection rate decreased from 5.63% in 2016 to 0.50% in 2022 (Fig 3). Using the evaluated point estimates of assay performance, diagnostic adjustment did not change the observed copro-ELISA positivity rate; therefore, the estimated decrease from 5.63% in 2016 to 0.50% in 2022 remained unchanged.

**Fig 4:**
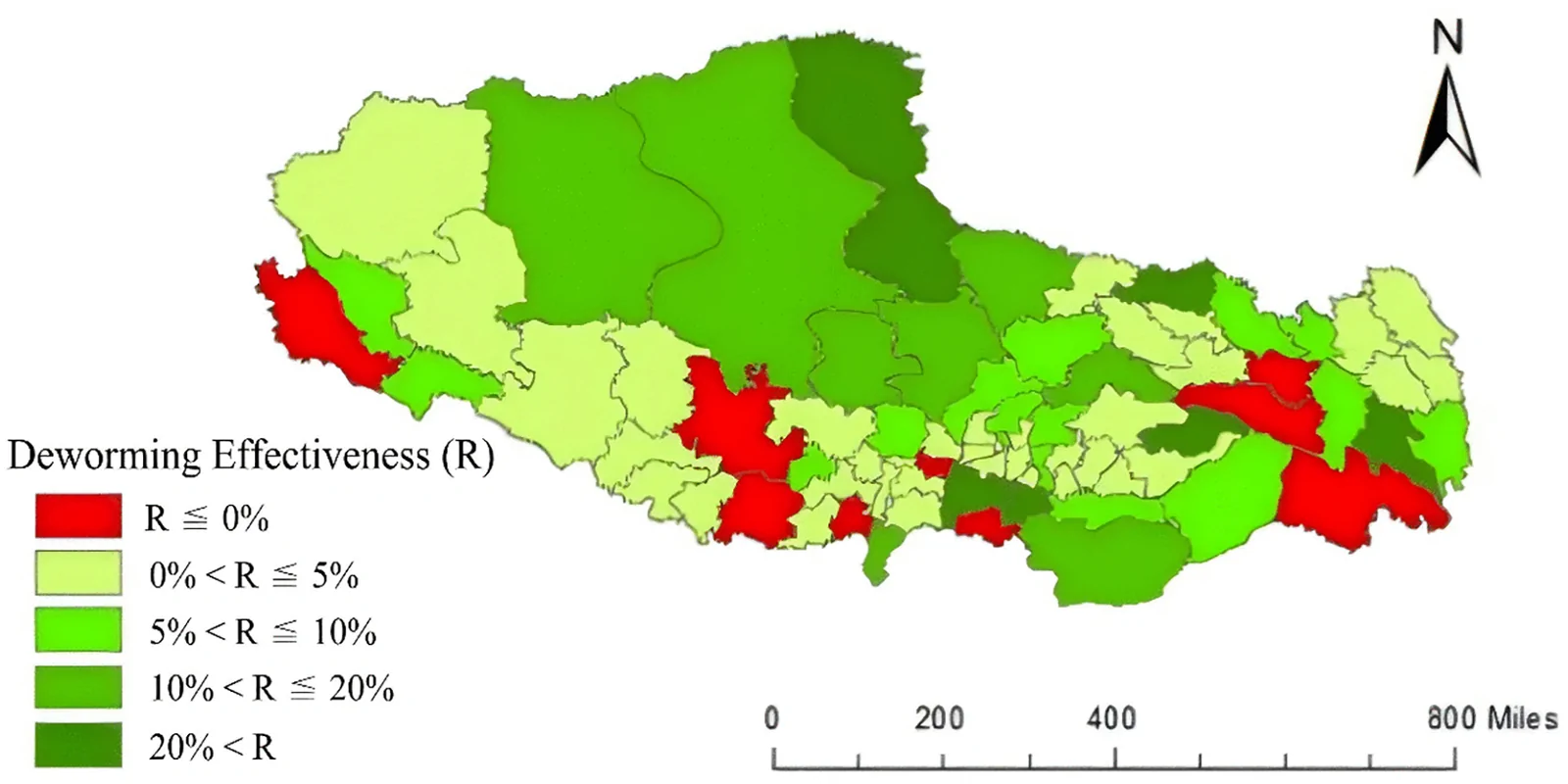
Dog deworming outcome reflected by the difference in canine infection rates between 2016 and 2022 across 74 counties in Xizang. The base layer is from *https://www.resdc.cn/DOI/DOI.aspx?DOIID=120* with credit to Resource and Environment Science and Data Center.

A comparative analysis of the rates in 2016 and 2022 was performed to examine the temporal changes. Fig 4 illustrates the spatial distribution of canine infection rate at the county level over time. In 2016, only 4 counties had infection rates below 1%, while 16 counties were in the (1%, 3%] range, another 16 in the (3%, 5%] range, and as many as 38 counties exceeded 5%. By 2022, a substantial decrease was observed, 38 counties had rates below 0.5%, 8 within (0.5%, 1%], 15 within (1%, 3%], 5 within (3%, 5%], and only 8 counties exceeded 5%. Among them, Zhongba County of Xigazê City reported the highest rate (8.88%), followed by Zayü County of Nyingchi City (7.76%) and Coqên County of Ngari Prefecture (6.19%).

Overall, most counties exhibited a decreasing trend in canine infection rate between 2016 and 2022. A total of 65 counties (87.8%) showed decreases compared with 2016, including 6 counties with decreases greater than 20%, 8 with decreases of (10%, 20%], 16 with decreases of (5%, 10%], and 35 with decreases of (0%, 5%]; 2 counties remained unchanged. However, 7 counties showed increased infection rates compared with 2016, including Zanda County of Ngari Prefecture (from 0% to 6.12%), Zayü County of Nyingchi City (from 3.00% to 7.76%), and Gamba County of Xigazê City (from 0% to 2.80%).

The canine infection rate has decreased significantly in Xizang. Further stratified analysis revealed that counties with a higher baseline infection rate in 2016 achieved greater relative reductions. All counties with an initial infection rate above 20% achieved decreases exceeding 80%, and seven of eight counties with a baseline rate of 10–20% also recorded decreases of more than 80%. These results highlight the substantial effectiveness of sustained deworming interventions across most areas (Table 2).

**Table 2:** Comparison of canine infection rates between 2016 and 2022 across 74 counties in Xizang.

Range of Infection Rate in 2016 (%)	Number of Counties with Increased Infection Rate	Number of Counties with Different Decreased Infection Rate (Magnitude of the Decrease, %)					Total Number of Counties
		Below 20	20-40	40-60	60-80	80 and Above	
0	2	0	0	0	0	2	4
(0, 1]	0	0	0	0	0	1	1
(1, 5]	5	1	3	3	5	15	32
(5, 10]	0	3	3	1	1	15	23
(10, 20]	0	0	0	0	1	7	8
(20, 41.3]	0	0	0	0	0	6	6
Total	7	4	6	4	7	46	74

Note: Counties are grouped by baseline canine infection rate in 2016. “Increased infection rate” indicates that the infection rate in 2022 exceeded that in 2016. Reduction categories indicate the percentage decrease relative to the 2016 baseline. Values represent numbers of counties.

### 3.3 Cost-outcome of dog deworming

**Fig 5:**
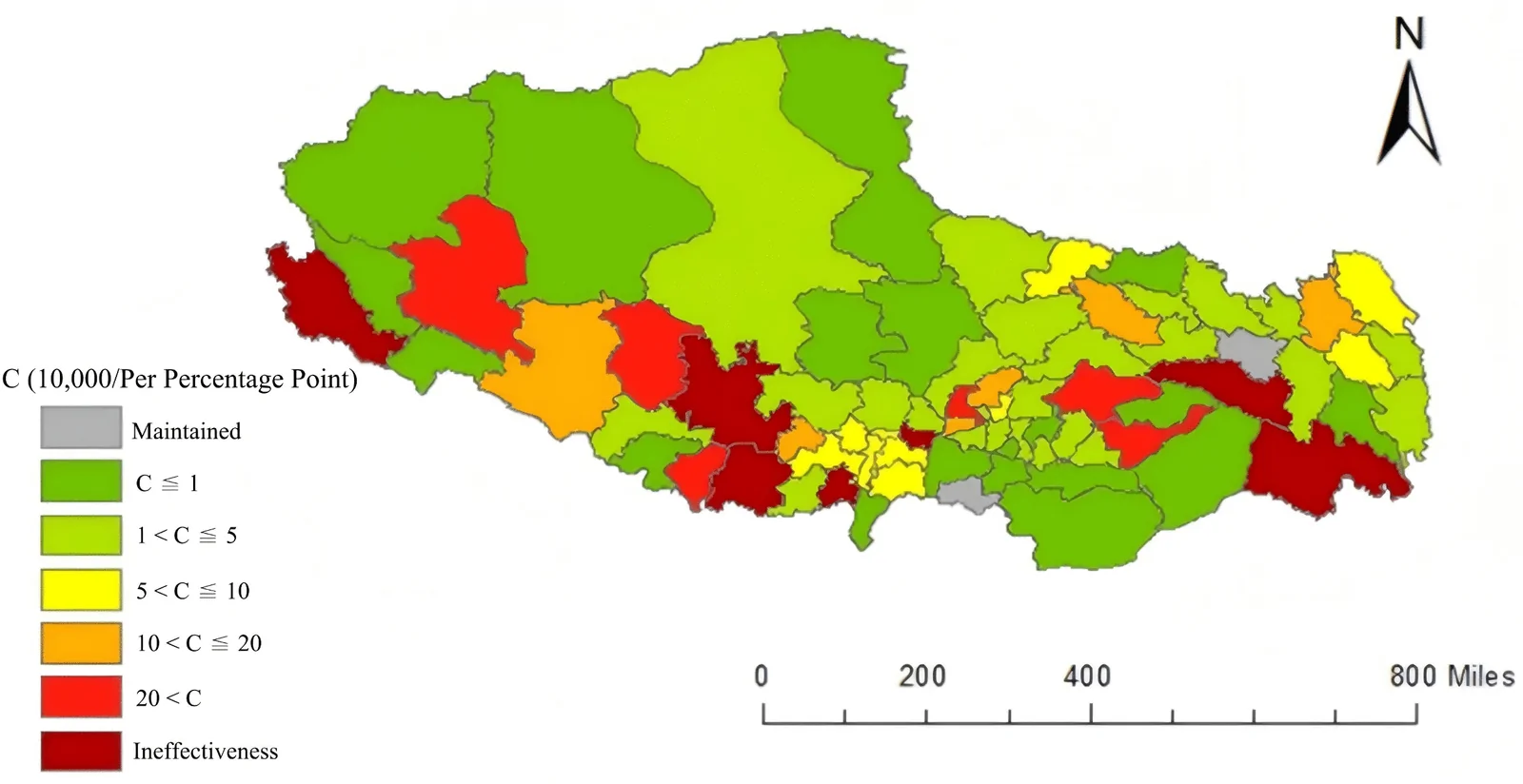
Cost of dog deworming per percentage point reduction in canine infection rate across 74 counties in Xizang, 2016-2022. The base layer is from *https://www.resdc.cn/DOI/DOI.aspx?DOIID=120* with credit to Resource and Environment Science and Data Center.

The cost required for each percentage-point reduction in canine infection rate was used as a comparative indicator of program efficiency across counties under a uniform control strategy. This metric may not be directly comparable across counties with very different baseline infection rates. A small absolute reduction in a low-prevalence county may appear inefficient despite successful maintenance of low transmission. Substantial heterogeneity in cost-outcome was observed across counties (Table 3, Fig 5), with the majority of counties achieving large reductions in canine infection rate at relatively low cost. Based on total cost and the reduction in canine infection rate from 2016 to 2022, 19 counties required less than 10,000 CNY for each percentage point reduction. The most favorable cost-outcome were observed in Comai County of Lhoka City (600 CNY per percentage point reduction), Cona County of Lhoka City (900 CNY per percentage point reduction) and Nagarzê County of Lhoka City (1,400 CNY per percentage point reduction). Another 25 counties required between 10,000 and 50,000 CNY per percentage point reduction, and only 6 counties exceeded 200,000 CNY per percentage point reduction, primarily located in Nyingchi City and Ngari Prefecture.

**Table 3:** Distribution of counties by reduction status and cost per percentage point reduction in canine infection rate in Xizang from 2016 to 2022.

Prefecture-level City	Counties with decreased canine infection rate: Cost per percentage point reduction (10,000 CNY)					Counties with no net change	Counties with increased canine infection rate	Total
	(0, 1]	(1, 5]	(5, 10]	(10, 20]	>20			
Lhasa	0	4	1	2	1	0	0	8
Xigazê	2	4	5	2	1	0	4	18
Lhoka	6	5	0	0	0	1	0	12
Qamdo	1	6	2	1	0	1	0	11
Nagqu	4	5	1	1	0	0	0	11
Nyingchi	2	1	0	0	2	0	2	7
Ngari Prefecture	4	0	0	0	2	0	1	7
Total	19	25	9	6	6	2	7	74

Note: Counties with decreased canine infection rates were further grouped by cost per percentage point reduction in canine infection rate (10,000 CNY). “No net change” indicates no change in canine infection rate between 2016 and 2022, and “increased canine infection rate” indicates a higher rate in 2022 than in 2016. Values are numbers of counties.

The majority of counties (44/74) required less than 50,000 CNY per percentage point reduction. However, 7 counties showed increased infection despite moderate investment, ranging from 34,000–378,000 CNY, primarily in Xigazê City, Nyingchi City, and Ngari Prefecture.

## 4. Discussion

The study assessed the cost, outcome, and cost-outcome of dog deworming in Xizang from 2016 to 2022. The canine infection rate in Xizang decreased from 5.63% in 2016 to 0.50% in 2022, representing a substantial reduction of 91.1% over the study period. Despite this achievement, substantial disparities in cost-outcome were observed across regions, highlighting the need to optimize both resource allocation and supervision of dog deworming implementation.

The dog registration rate in Xizang increased from 81.6% in 2016 to 96.2% in 2022. Overall, this increasing trend indicates improvement in the registration system and management coverage, although fluctuations were observed in some years. This may reflect recent years of efforts in stray dog sheltering, alongside educational campaigns aimed at changing herders’ dog-keeping practices [16]. Progress in dog registration has also facilitated the implementation of dog deworming measures.

Regarding deworming cost, expenditures increased from 2016 to 2018, peaking in 2018, then decreased in subsequent years. Spending was positively correlated with the deworming compliance rate, suggesting that financial input may support program implementation. However, since 2020, the deworming compliance rate in Xizang has shown a decreasing trend, decreasing to 77.20% in 2022. This decrease may be associated with the phased nature of the control program. In 2016, a comprehensive epidemiological survey revealed that all counties in Xizang were endemic for echinococcosis, followed by a region-wide assessment of control activities in 2017. From 2018 to 2020, Xizang launched a three-year intensive control campaign, which effectively enhanced public awareness and participation in disease prevention. Nevertheless, after the conclusion of the campaign, possible complacency arising from earlier success and a reduction in health education efforts may have contributed to a certain relaxation of control measures, leading to a decrease in deworming compliance rate. This trend highlights the importance of sustained health education and behavioral interventions in maintaining the long-term effectiveness of dog deworming programs [22]. Expanding health education on echinococcosis transmission, the importance of dog deworming, and appropriate supervision of deworming practices may improve public cooperation and compliance.

Substantial reductions in canine infection rates were observed from 2016 to 2022: 87.8% (65/74) of counties showed decreases, and 46 counties achieved reductions of more than 80%. However, 7 counties showed increases. The reasons for increased infection rates in a small number of counties remain uncertain. Among these, 2 counties increased from previously reported 0% infection rates in 2016, 2 showed only slight increases, and 3 experienced relatively large increases. These findings suggest that, although the overall control effect was favorable, a small subset of counties still showed adverse control outcomes. The reasons for increased infection rates in a small number of counties remain uncertain. Potential explanations include variation in program implementation, local transmission dynamics, sampling variability, and measurement uncertainty. In such areas, prompt field investigation is needed to identify the causes and guide targeted interventions. The 2 counties that increased from previously reported 0% infection rates also warrant attention. This pattern may reflect cross-transmission from neighboring endemic areas, although the evaluated specificity of the kit was high, diagnostic uncertainty cannot be completely excluded when interpreting low copro-ELISA positivity rates, as reflected by the confidence interval of the specificity estimate [16]. However, given that echinococcosis remains endemic throughout Xizang, renewed detection in counties with previously reported 0% infection rates is not entirely unexpected. This is especially relevant in remote, high-altitude pastoral areas, where dog populations are relatively large and management is more difficult [23]. In such settings, inadequate health resources, limited public awareness, and poor dog management may further contribute to unfavorable outcomes [24,25]. Continued efforts should focus on strengthening supervision of dog deworming implementation, promoting dog tethering, and ensuring safe disposal of canine feces after deworming in affected counties. Although standardized testing and sample-review procedures were implemented to improve the reliability of surveillance results, ELISA-based detection still has certain limitations. Some truly positive samples may have been misclassified as negative and not selected for review, and minor variation between production batches of diagnostic reagents may also have introduced limited measurement uncertainty.

The cost-outcome analysis showed relatively favorable program efficiency in most counties, especially in Lhoka City, where the cost per percentage point reduction in canine infection rate was lower than 10,000 CNY. These findings indicate that, under the strong commitment of the Xizang government and the support of the Central Government’s Transfer Payment Project for Echinococcosis Control, dog deworming has achieved relatively good program efficiency in many counties. However, a few regions showed high investment but limited reduction in canine infection rate, alongside persistently high infection rate, implying the current measures are not adequately controlling transmission. Potential causes include weak implementation of preventive measures or non-standardized deworming practices. Promoting routinely monitoring and evaluating deworming outcome, and improving the quality of local deworming operations are likely to improve program efficiency. These findings are consistent with international and national experiences from long-term echinococcosis control programs, which have consistently shown that regular, high-coverage dog deworming is a critical component of successful transmission control, while sustained implementation and integration with complementary measures are essential for maintaining long-term gains [26,27].

It should be noted that the reduction in canine infection rates was likely the result of the combined effects of multiple measures implemented under the integrated echinococcosis control program, including dog deworming, dog management, stray dog sheltering, and health education. Within the Central Government’s Transfer Payment Project for Echinococcosis Control, dog deworming has consistently been one of the key source-control measures. From 2006 to 2018, monthly deworming of dogs was required in the most severely endemic counties, and since 2019, monthly deworming has continued in counties with relatively heavy endemicity, while quarterly or semiannual deworming has been adopted in areas with lower counties. Because counties in Xizang share broadly similar geographic and ecological conditions in the hinterland of the Qinghai-Tibet Plateau, the influence of natural factors on county-level differences may have been relatively limited, despite echinococcosis being a natural focal disease. In addition, dog management, stray dog sheltering and health education were implemented across Xizang under broadly similar policy requirements and control frameworks, which may have reduced variation in their effects across counties. However, because this was an ecological observational analysis based on aggregated county-level data. Therefore, the observed reductions in canine infection should be interpreted as being associated with dog deworming as part of integrated control measures, rather than as evidence of the independent effect of dog deworming alone.

Dogs are the definitive hosts of Echinococcus and play a critical role in increasing the transmission risk to humans and livestock [28,29]. For this reason, dog management and deworming are important measures for reducing human exposure risk in Xizang, where all counties are categorized as level I or II echinococcosis-endemic areas in 2016 [10,25,30]. Praziquantel remains the drug of choice for dog deworming, and its administration should be standardized according to dog weight [20,30]. In addition, proper record keeping, routine drug distribution, and safe disposal of canine feces after deworming should be strengthened to lower transmission risk and help interrupt transmission [31,32].

Several limitations should be noted. First, this was an ecological observational study based on aggregated county-level data. No control group was included, and no causal modelling was performed; therefore, the findings are programmatic and observational, not proof of causality. Second, although the evaluated point estimates of sensitivity and specificity were 100%, the confidence intervals indicate residual uncertainty. Therefore, a small degree of misclassification cannot be completely excluded under field conditions. Potential false-negative results may also not always have been selected for review. Third, the cost-outcome indicator used in this study may be influenced by baseline infection levels. Counties with low initial prevalence may appear less efficient despite successful maintenance of low transmission. Therefore, comparisons should be interpreted with caution. Finally, no formal adjustment for inflation was performed. Although the cost estimates were derived from observed annual program budgets determined under standardized governmental procurement and budgeting rules, costs across years may still not be perfectly comparable.

## 5. Conclusion

In summary, dog deworming, as part of a sustained control program was associated with reductions in canine infection rate and should be sustained continuously with high quality. The study showed a significant decrease in the canine infection rate during 2016–2022, although substantial regional differences in cost-outcome remained. These findings provide a basis for optimizing resource allocation and supervision of dog deworming implementation in Xizang, and may also offer valuable reference for other endemic areas according to local conditions.

## Abbreviations

CE: cystic echinococcosis
AE: alveolar echinococcosis
DALYs: disability adjusted life years
R: deworming outcome.
